# The Association Between Social Desirability and Competitive Anxiety in Young Football Players with Different Qualifications

**DOI:** 10.11621/pir.2024.0308

**Published:** 2024-09-15

**Authors:** Murad B. Sultanov

**Affiliations:** a *Academician A. Garayev Institute of Physiology, Ministry of Science and Education of the Republic of Azerbaijan, Baku, Azerbaijan*

**Keywords:** football, social desirability, competitive anxiety, male, team sports

## Abstract

**Background.:**

Sports performance anxiety is understood as a tendency to respond with cognitive or physical anxiety in competitive situations where the athlete’s performance can be assessed.

**Objective.:**

To investigate the role of social desirability and competitive anxiety in male football players with varying levels of skill. The study had two main objectives: first, to assess the levels of social desirability and competitive anxiety in two different groups, and second, to compare the levels of social desirability and competitive anxiety between highly skilled and less skilled players and explore their relationship.

**Design.:**

Participants were divided into highly skilled (*n* = 39) and less skilled (*n* = 39) football players. The Eysenck Personality Questionnaire was used to assess the participants’ social desirability bias. The Sport Competition Anxiety Test assessed the level of competitive anxiety. The *t*-test for independent samples was used to compare social desirability and competitive anxiety between the groups. A linear regression model was used to determine if social desirability could predict anxiety levels among the players.

**Results.:**

The *t*-test showed that highly skilled players have a lower level of competitive anxiety than less skilled participants. However, social desirability did not show a statistically significant difference between the highly skilled and less skilled football players. The regression analysis showed a statistically significant association between social desirability and competitive anxiety (inverse relation) in both groups.

**Conclusion.:**

Highly skilled football players demonstrated less competitive anxiety than less skilled ones. Lower levels of competitive anxiety are associated with higher social desirability among football players in both groups.

## Introduction

The Football World Cup, held in Qatar in 2022, once again confirmed the thesis that in elite sports, the personal characteristics of players, and in particular, their competitive anxiety, play an important role in achieving high results (Sultanov & Ismailova, 2019). Several studies have demonstrated differences in characteristics between highly skilled and less skilled players (de Gouvêa et al., 2017; Williams, 2000; Wright et al., 2013). However, for some personality traits, there have been few studies of these differences. At the same time, the relationship between the personal characteristics of athletes and their performance remains a pressing problem in team sports. Players’ personalities are currently under active study due to the psychophysiological demands on their bodies and the impact on their sports performance. The issue of tolerance for various competitive situations and its correlation with personality traits and competitive anxiety among team sports players is being addressed (Sultanov, 2023). Sports performance anxiety (Grossbard et al., 2007) has been conceptualized as “a predisposition to respond with cognitive and/or somatic state anxiety to competitive sport situations in which the adequacy of the athlete’s performance can be evaluated” (Smith, Smoll, & Wiechman, 1998, p. 107). R. Martens notes that athletes with higher levels of competitive trait anxiety tend to perceive competitive situations as more threatening than athletes with lower levels of anxiety (Martens et al., 1990). Several more recent studies have explored the role of anxiety in sporting performance (Franklin et al., 2015; Moore et al., 2013; Wilson et al., 2007). Anxiety can have a positive, negative, or no effect on performance, depending on the individual’s level of anxiety and the attentional demands of the task. The influence of pre-competitive anxiety on athletes’ performance depends largely on the interaction of the athlete’s temperament and the competition situation.

Previous research has indicated that athletes in team sports exhibit different personality traits than those in individual sports (Allen et al., 2013), and that anxiety levels in team and individual sports may also differ (Eagleton et al., 2007). Team and individual sports are conceptually different, with team sports relying on collaboration and social interaction to a greater extent (Wold et al., 2013). Team players are generally more extroverted, anxious, and dependent, but less sensitive and imaginative than individual sports players (Cox, 1998). Concurrently, limited research has investigated social desirability factors in the context of competitive anxiety, and their relationships within team sports.

In the *Encyclopedia of Social Measurement,* T. Graeff (2005) mentioned that social desirability bias is linked to other personality factors such as anxiety, achievement, motivation, and self-esteem. Physical activity is considered socially desirable behavior (Motl et al., 2005). Moreover, social desirability bias is included in football development programs (Feichtinger & Höner, 2014). Athletes with high social desirability described greater coach support than those with low social desirability, among male and female high school tennis players, as measured by the Sport Competition Anxiety Test (SCAT) (Ryska, 1993). Thus, people may respond in a way that they think is socially desirable, which can affect how anxious they feel in sports situations. In team sports, especially football, social desirability has not been studied much, and research in this area has been pretty poor (Smith et al., 2002).

The present study aimed to test the association between social desirability and competitive anxiety among football (soccer) players with different qualifications. The first purpose of the study was to reveal the level of social desirability and competitive anxiety in two groups of interest, and the second purpose was to compare social desirability and competitive anxiety levels between highly skilled and less skilled players and to investigate their relationship.

In football, as a highly competitive game, levels of social desirability and anxiety might differ between highly skilled and less skilled football players. The hypothesis is that a highly competitive game such as football has a difference in personality traits between highly skilled and less skilled players.

## Methods

### Participants

The participants were male football players 17–21 years old: 39 highly skilled players (M = 18.3, SD = 1.0) and 39 less skilled players (M = 18.4, SD = 1.1). The highly skilled football players had more training hours per week (9 vs. 4.5, respectively) and a more intensive training process than less skilled players did. The highly skilled players participated in the national youth football championship, and some players were recruited for the national youth football teams. The less skilled players had 4.5 football training hours per week at Sports University. This group engaged in practice of other sports as individuals and teams, with a minimum of three training hours per week. In addition, the less skilled players were involved in competition with amateur football teams (2–5 competition hours per month). The classification of football players into highly skilled and less skilled categories has been established in the literature (de Gouvêa et al., 2017). Participants had normal hearing and vision and no psychiatric or neurological disorders.

### Measures

#### Social Desirability Scale

Social desirability was measured using Eysenck’s Personality Questionnaire (EPQ) (Eysenck & Eysenck, 1975). The EPQ was available for both languages (Azerbaijan and Russian) that were used in this study. The social desirability test is carried out using 25 questions on the EPQ. The psychometric estimation proposed by Eysenck’s Personality Theory has been widely used and well established in several studies (Barrett et al., 1998; Razumnikova, 2004). In addition, a previous study mentioned that component ‘A’ in the EPQ lie (social desirability) scale functions as an index of socially conforming behavior (Francis, 1991).

#### Competitive Anxiety

The competitive anxiety of participants was evaluated by the Sport Competition Anxiety Test (SCAT), a 15-item scale that measures trait anxiety among sports performers (Martens, 1977). Respondents were required to indicate their agreement with each item by selecting their answer from *‘rarely’, ‘sometimes’,* and ‘*often’* (three-point Likert scale)*.* Five of the 15 items in the SCAT questionnaire are ‘buffer’ questions (Iwuagwu et al., 2021). The scores on this test may vary from 10 points to 30 points. Various general anxiety inventories have been correlated with the test, demonstrating its convergent validity (Lavallee et al., 2012). For the Russian language, this test was translated and validated by Y. Hanin (Feltz et al., 1982); for the Azerbaijani language, this manipulation was done by the author using the Russian model.

### Procedure

To assess social desirability and competitive anxiety, participants completed the EPQ and the SCAT after training at home and gave the forms to the researcher. In the first stage, players completed the EPQ and then the SCAT. The researcher allowed each participant nearly one week to complete both questionnaires. Data from tests and results were completed and analyzed in approximately six months.

Before the selection of participants, the researcher talked with coaches and supervisors to support him in choosing players for research in both groups. Highly skilled players were recruited for study according to actual performance before competition season (second part). Requirements for participation in this study were competitive practices, a high team position in the tournament after the first part, and membership in national youth teams (if possible). Less skilled players were recruited for the study from the Sports University, and had trained in the Football specialty. The study began in October 2015 and concluded in April 2016. The researcher obtained permission from the management of the football clubs to study the players. University students were given course credits for participating in the study. Each participant gave verbal consent for the testing. Data were collected before the COVID-19 lockdowns in Azerbaijan.

### Statistical Analysis

The Shapiro-Wilk (SW) test was used to verify the normality of the data and assume normality for residual errors among participants. The study employed a *t*-test for independent samples to compare the social desirability and competitive anxiety between the two groups. A linear regression model was used to analyze social desirability as a predictor of competitive anxiety by cross-sectional study. The level of significance was set at *p* < .05. Statistical analysis of the data was performed using SPSS Statistics for Windows, v.23.0. Armonk, NY: IBM Corp. (USA) and ‘Statistics Kingdom’.

## Results

The study found that both of the groups demonstrated approximately equal data on the social desirability scale: highly skilled players (M = 13.87; SD = 5.09; Cronbach’s α = .57) and less skilled football players (M = 14.28; SD = 5.25; α = .43). The SCAT showed a low level of competitive anxiety in highly skilled players (M = 15.54; SD = 3.05; α = .43) compared to the less skilled football players (M = 17.23; SD = 3.59; α = .44). Thus, the *t*-test for independent samples demonstrated a statistically significant difference (*p*= .03). This data revealed that highly skilled football players have a lower level of competitive anxiety than less skilled participants. On the other hand, social desirability did not show a statistically significant difference between the two groups (see *Table 1*).

**Table 1:** Descriptive Statistics and Analysis of Differences Between Highly and Less Skilled Players

	Highly Skilled			Less Skilled			*t*-test for Equality of Means				
Scale	Mean	*SD*	α	Mean	*SD*	α	95% CI		*t*	*df*	*p*
Social desirability	13.87	5.09	.57	14.28	5.25	.43	–2.74	1.92	–.35	76	.73
Competitive anxiety	15.54	3.05	.43	17.23	3.59	.44	–3.19	–.19	–2.24	76	.03

*Notes. The t-test illustrated that the two groups have differences in the competitive anxiety scale. SD = standard deviation. α = Cronbach’s alpha. CI = confidence interval. t = t-value. df = degrees of freedom. p = significance.*

**Figure 1.**
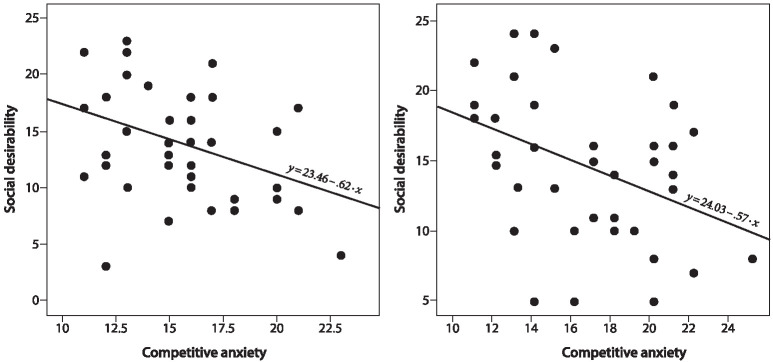
The relationship between social desirability and competitive anxiety among highly skilled players (at the left) and less skilled players (at the right)

Social desirability is a predictor of competitive anxiety in both groups. This relationship includes both highly skilled and less skilled players, each with approximately equal values (see *Figure 1*). A regression analysis suggests that the relationship between social desirability and competitive anxiety in the two groups of players has a negative slope (inverse relation). Consequently, when social desirability is higher, the competitive anxiety among players is lower, and conversely. The summary of regression analyses for the group of highly skilled players is as (*F*_(1,37)_ = 5.85; *β* = –.22; *p* = .02), and for the group of less skilled footballers as (*F*_(1,37)_ = 6.49; *β* = *–*.26; *p* = .02) (see *Table 2*).

**Table 2:** Results of Regression Analyses for Competitive Anxiety in Two Groups of Football Players

Parameter	F_(1,37)_	R^2^	*t*	β	95% CI		Correlation	Power	*p*
*Highly skilled players*									
Social desirability	5.85	.14	–2.42	–.22	15.87	21.34	–.37	.66	.02
*Less skilled players*									
Social desirability	6.49	.15	–2.55	–.26	17.81	24.19	–.39	.66	.02

*Notes. F = f-value. R2= R-squared. β = beta coefficient.*

## Discussion

This study found that highly skilled players have lower competitive anxiety than less skilled football players. This is in line with other evidence, which demonstrated that football players were lower on the neuroticism-anxiety scales than general university participants (O’Sullivan et al., 1998). In addition, the low scores for competitive anxiety among highly skilled football players compared to less skilled players are consistent with evidence that regular exercise reduces anxiety (Eagleton et al., 2007; McKelvie et al., 2003). Consequently, experience and practice enable players to identify the individual methods that reduce competitive anxiety (Mottaghi et al., 2013). For instance, other research (Cartoni et al., 2005; Pears, 2007) illustrated that as athletes age, their anxiety decreases. This finding is consistent with our own, suggesting that increased competitive experience and training hours may reduce competitive anxiety. These factors suggest that team sports may cause personal changes in individuals over their sporting lifetimes. During sports activities, monitoring the helpfulness of players to their team can be used to determine the reorganization of individual-typological traits of highly skilled players compared to less skilled football players.

On the other hand, these results indicate that the skill level of football players does not affect the predictive power of social desirability for competitive anxiety among participants. Social desirability may decrease anxiety in competition through the suppression bias of individual responsibility. People with high trait anxiety tend to notice more threat-related information due to a cognitive bias (Martens et al., 1990). Therefore, players will be inspired by teamwork in contrast to self-estimation. Social desirability bias may be particularly useful for players with high levels of competitive anxiety. This behavior may have an effect on the performance. Some evidence suggests that high levels of competitive anxiety are related to poor performance (Scanlan et al., 2005; Smith & Smoll, 1991). Thus, the inverse correlation between social desirability and competitive anxiety potentially indicated this association. According to other studies, highly successful athletes have positive thoughts, better concentration, are more task-oriented, and have lower levels of anxiety (Ahmad & Safdar, 2020). In team sports, social interaction can influence overall team performance (Verburgh et al., 2014). Another study showed that social desirability was not connected with neuroticism; however, this study was conducted on non-sport subjects (Davies et al., 1998). By contrast, in this study, both groups practiced sports. Another study demonstrated that social desirability is more strongly correlated with pre-competitive anxiety when an individual plays soccer (football) professionally rather than at the varsity level (Smith et al., 2002).

Accordingly, this study revealed an association between social desirability and competitive anxiety among football participants. This suggests that social desirability has a connection to competitive anxiety and may be associated with player performance. However, the relationship between social desirability and competitive anxiety may vary across different sports. According to these results, in football, social desirability is approximately equal among highly skilled and less skilled football players. Only the level of competitive anxiety indicated a statistically significant difference between the two groups. In the group of highly skilled players, the level was lower. The results have not illustrated differences in the relationship between social desirability and competitive anxiety in both groups, despite the contrast in the anxiety level. Thus, in future studies that will explore the relationship between social desirability and personality traits, it may be reasonable to use samples that demonstrate a wide range of abilities in sports. If a difference between social desirability or personality traits is observed, it could provide some evidence of the distinction between highly skilled and less skilled participants.

In addition, it has been found that gamma rhythm in the prefrontal cortex is associated with social desirability, which is reflected in the independence of decision-making or selective behavior modification among football players (Sultanov, 2020). The critical role of social desirability in the frame of relationship with brain activity in the frontal lobe is also supported in a study by O. Razumnikova (2004) with female participants. Furthermore, in a previous study, we determined the relationships between social desirability, type of temperament (with a tendency towards extraversion), and anxiety among football participants (Ismayilova & Sultanov, 2023).

A similar study (Grossbard et al., 2007) mentioned that “Social desirability response set may also influence another widely studied variable in sport, namely anxiety. Sport performance anxiety has been conceptualized as ‘a predisposition to respond with cognitive and/or somatic state anxiety to competitive sport situations in which the adequacy of the athlete’s performance can be evaluated’ (Smith, Smoll, & Wiechman, 1998, p. 107)”. This research has expanded this hypothesis and revealed that not only is social desirability a response, but it may also be linked with players’ behavior and, accordingly, has a connection with anxiety in competitions on different levels. These factors may have a relationship with the performance and results of football players.

The present results confirmed one out of two working hypotheses: highly skilled players have a lower level of competitive anxiety than less skilled football players; however, social desirability in the two groups showed approximately equal results. This may indicate that the level of social desirability does not change during lifespans.

## Conclusion

Highly skilled football players showed lower competitive anxiety than less-skilled participants. However, the study did not reveal any differences in the level of social desirability between the two groups. The data show that the skill level of football players is not associated with social desirability’s prediction of competitive anxiety and is linked to consistent participation in sports activities.

Therefore, if the level of social desirability among players increases, their competitive anxiety decreases. Social desirability traits may have a positive role in excessive anxiety among team sports athletes. One effective way to achieve important goals is to foster social desirability behavior among football players within a team. This might include benefits for a football team, such as comradery and consonance.

## Practical Implications

“The developmental hypothesis” (Elman & McKelvie, 2003) declared that continuous sports practice on the professional level causes personality changes and, respectively, the differences between highly skilled and less skilled football players. The specifications for reorganizing individual-typological traits of highly experienced players, compared to those who are less skilled, can be clarified by monitoring their contributions to team goals during sports activities. Thus, trainers need to use different approaches to regulating football players’ behavior during various situations in the match. This can help determine the best way to balance individual personalities with the team’s priorities to achieve short-term and long-term goals. 

## Limitations

The study involved a small number of participants, and therefore, future research should endeavor to replicate and expand upon these findings using a larger sample. In addition, this research was conducted with a relatively young age group of sportsmen, and future studies need to check these results on adult athletes.

Moreover, social desirability and competitive anxiety traits will be interesting to observe in participants in individual sports. Further studies could investigate the correlation between social desirability and other personality traits in team and individual sports.
